# Impact of Bariatric Surgery on Ghrelin and Obestatin Levels in Obesity or Type 2 Diabetes Mellitus Rat Model

**DOI:** 10.1155/2014/569435

**Published:** 2014-02-10

**Authors:** Donglei Zhou, Xun Jiang, Weixing Ding, Dingyu Zhang, Lei Yang, Chengzhu Zhen, Liesheng Lu

**Affiliations:** ^1^Department of General Surgery, Shanghai Tenth People's Hospital of Tongji University, Shanghai 200072, China; ^2^August First Physical Culture and Sports Team, Haidian District, Beijing 100091, China; ^3^Department of General Surgery, Changhai Hospital of the Second Military Medical University, Shanghai 200433, China

## Abstract

We aimed to evaluate the therapeutic efficacy on weight control by different bariatric surgeries and investigate the ghrelin and obestatin changes after these surgeries in obesity and nonobese type 2 diabetes mellitus (T2DM) rats. Obese rats were randomly assigned to receive sleeve gastrectomy (SG, *n* = 8), minigastric bypass (MGBP, *n* = 8), roux-en-Y gastric bypass (RYGBP, *n* = 8), and sham operation (SO, *n* = 4). Another 4 rats served as control. Besides, Goto-Kakisaki (GK) rats were also randomly divided into similar groups except for total gastrectomy (TG, *n* = 8) group. The results showed that in obese rats, weigh loss in RYGBP group was similar to that in MGBP group but larger than that in SG group. Ghrelin significantly increased in RYGB group, but obestatin increased in MGBP group. Ghrelin/obestatin ratio significantly decreased in SG group. In GK rats, weight loss was most obvious in TG group. Postoperatively, ghrelin was significantly increased in MGBP and RYGB groups but decreased in TG group. Obestatin also showed an increase in MGBP and RYGB groups. Ghrelin/obestatin in TG group decreased significantly. In conclusion, RYGB and MGBP may be more suitable for obese rats, but TG may be the best strategy for T2DM rats to control weight with different mechanisms.

## 1. Introduction

Obesity and type 2 diabetes mellitus (T2DM) have currently become 2 of the most common chronic diseases globally [1]. According to World Health Organization estimates, the numbers of people with obesity and T2DM worldwide will reach 700 million and 300 million, respectively, by 2015 [2]. Both diseases are closely associated with excessive food intake and a sedentary lifestyle. Thus, lifestyle modification (including diet, physical activity, and behavior therapy) is usually suggested to promote weight loss and prevent the disease development [3]. However, none of these treatments has long-term success because it is difficult to maintain healthy eating and exercise behaviors over time [4].

Recently, accumulating evidence has demonstrated that bariatric surgery is a more effective way to produce greater and sustained body weight reduction (weight loss of 50% to 75% of excess body weight) compared with nonsurgical treatments [5, 6]. The commonly used bariatric surgeries include roux-en-Y gastric bypass (RYGB), minigastric bypass (MGB) and sleeve gastrectomy (SG). Lee et al. reported that participants assigned to RYGB lost more weight than the SG group [7]. In addition, they also found that MGBP was superior to RYGB in lowering the body mass index (BMI) and increasing the excess weight loss [8, 9]. These findings indicate that the effectiveness sequence was MGB > RYGB > SG. However, Padwal et al. obtained the inconsistent conclusions: differences in BMI levels from baseline are as follows: MGB [−11.3 kg/m^2^], SG [−10.1 kg/m^2^], and RYGB [−9.0 kg/m^2^] [10]. Thus, further effect study is still needed.

Furthermore, it is hypothesized that the preliminary encouraging results with these methods in weight loss may be attributed not only to the restriction of the gastric capacity, but also to the drastic changes in hormone secretion (ghrelin and obestatin), leading to large decreases in appetite and food intake [11]. However, there are controversial results about the hormone changes after surgery. Some studies reported a significant decrease in ghrelin and obestatin levels after surgery [12–15], while others, increase [16–18] or no change [12]. Importantly, different surgeries achieve different influence, for example, plasma obestatin concentrations were shown to be significantly increased in patients after SG but without any alteration after GB [19]. Thus, further mechanism study is still required.

The aim of this study was to further evaluate the therapeutic efficacy on weight control by different surgeries and investigate the changes of ghrelin and obestatin before and after these surgeries in obesity and non-obese T2DM rat models.

## 2. Materials and Methods

### 2.1. Experimental Animals and Grouping

All animal studies have been approved by China Ethics Committee and performed in accordance with the ethical standards. A total of 70 specific pathogen-free (SPF) grade male Sprague-Dawley (SD) rats aging 6 weeks old with an average weight of 210 ± 20 g and 40 SPF grade male Goto-Kakisaki (GK) rats (a non-obese model of spontaneous T2DM) aging 9 weeks old with an average weight of 340 ± 20 g were purchased from Shanghai Slyke Laboratory Animal Corp. (Certificate no.: SCXK 2008-0003). All animals were weighed and bred to adapt to the environment for 1 week. Then SD rats were randomly divided into 2 groups to be fed with a normal diet (*n* = 35) and high-lipid diet (*n* = 35) for 7 weeks. If the weight of rats in obese group exceeded 20% of average weight of rats in normal group, animal model of obesity was considered to be established successfully. Subsequently, obese rats were randomly assigned to receive SG (*n* = 8), MGBP (*n* = 8), YGBP (*n* = 8), and sham operation (SO, *n* = 4). Four rats served as control without any treatment (NC, *n* = 4). Another 6 rats were used as supplements in case of an unexpected death in surgery. GK rats were also fed with a high-fat diet for 10 weeks and then randomly divided into SG group (*n* = 8), MGBP (*n* = 8), YGBP (*n* = 8), total gastrectomy (TG) group (*n* = 8), SO group (*n* = 4), and NC group (*n* = 4). All procedures were conducted according to the Guide for the Care and Use of Laboratory Animals.

### 2.2. Surgical Procedures

The rats were fasted for 7 h preoperatively for upper gastrointestinal surgery, but 24 h for lower gastrointestinal surgery. Glucose can be added in animal's drinking water to supplement energy and increase their tolerance for anesthesia. The rats were anesthetized with 1% sodium pentobarbital solution (30–50 mg/kg) injected intraperitoneally into rats for 60–120 min. Antibiotic prophylaxis was applied with intramuscular 5% cefradine (0.02 g/kg). Sleeve gastrectomy (SG) involves the creation of a reduced stomach lumen with a 48-Fr bougie at the lesser curvature through the removal of gastric tissues along the greater curvature from the fundus to the antrum [20]. In MGBP surgery, a long gastric tube was created approximately 1.5 cm to the left of the lesser curvature from the antrum to the angle of His. A loop gastroenterostomy was created with the small bowel about 20 cm distal to the ligament of Trietz [8]. In YGBP group, the dissection began directly on the lesser curvature of the stomach, and a gastric pouch of about 20% of the total gastric volume was created. The pouch was anastomosed to a Roux limb of jejunum created by division of the jejunum 15 cm distal to the ligament of Treitz and anastomosing the afferent biliopancreatic limb to the jejunum 15 cm distally [21]. A total gastrectomy (TG) was performed with the removal of the entire stomach and end-to-end anastomosis of the esophagus and duodenum. Sham operated (SO) controls received manipulation of the stomach and a transverse enterotomy at the same position of the proximal jejunum; however, this was reclosed without forming an anastomosis. All surgeries were performed with the first author. The surgery time was 40–60 min for SG and TG, but 60–90 min for MGBP and YGBP. Postoperatively, animals were given ad libitum access to water and a high-fat diet. Additional doses of fluid infusion (glucose) were also administered to accelerate recovery.

### 2.3. Body Weight Measurement

Electronic scale was used to record body weight of rats every day under their quiet and awake condition until 12 weeks or 20 weeks postoperatively for obese rats, but 5 weeks postoperatively for GK rats.

### 2.4. Ghrelin and Obestatin Measurement

Venous blood from angular vein was collected into potassium/EDTA-coated tube containing 0.6 TIU/mL aprotinin. Samples were then centrifuged at 1600 g for 15 min at 4°C and plasma kept at −80°C for later analyses. Fasting blood glucose was determined by using spectrophotometer. Ghrelin and obestatin plasma levels before, 4 weeks, and 12 weeks after operation in obesity rats and before, 2 weeks, and 5 weeks after operation in GK rats were determined by radioimmunoassay (RIA, Phoenix Pharmaceuticals, Inc., USA) according to the manual descriptions.

### 2.5. Statistical Analysis

All data were analyzed by SPSS 13.0 software and were displayed by mean ± SD. Paired Student's *t*-test was used to compare data before and after the operation, Student's *t*-test or *t*′ test was calculated according to the homogeneity of variance detected by *F* test, and analysis of covariance (ANCOVA) further corrected the differences among groups before operation. Pearson's correlation coefficient was used to test the correlation among the indicators. *P* < 0.05 was considered statistically significant.

## 3. Results

### 3.1. Obesity and T2DM Rat Models

**Figure 1:**
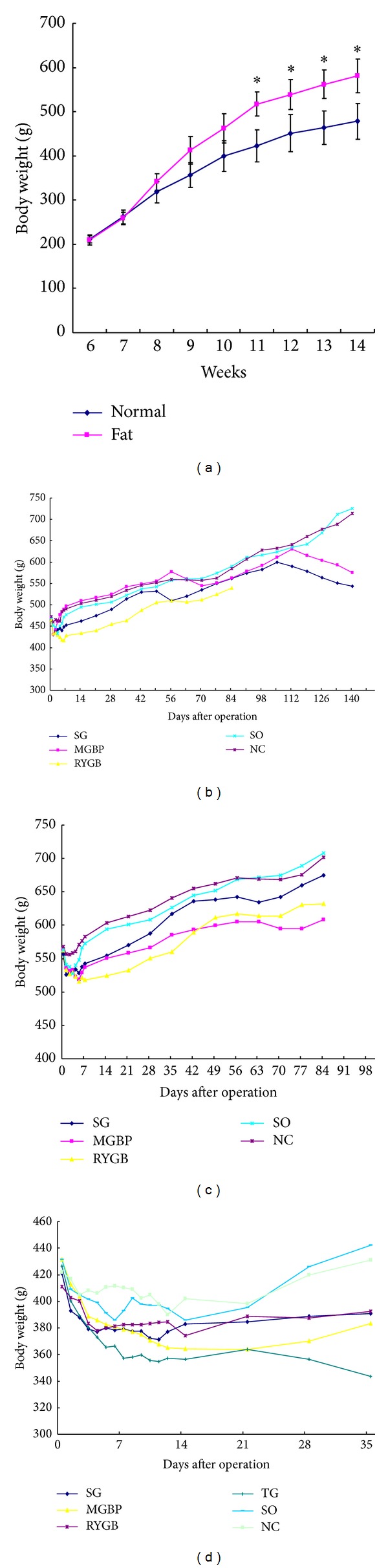
Body weight changes after different diets feed. (a) Preoperative body weights of rats with a normal diet or high-lipid diet; (b) postoperative body weight in normal rats; (c) postoperative body weight in obese rats; (d) postoperative body weight in Goto-Kakisaki (GK) rats. *Compared with the body weight of rats before experiment. SG, sleeve gastrectomy; MGBP, minigastric bypass; RYGBP, roux-en-Y gastric bypass; SO, sham operation; NC, normal control; TG, total gastrectomy.

After being fed with a high-fat diet for 3 weeks, body weight of obesity rats group was significantly higher than that of the control group. In the 7th week, obesity rat model had been successfully established (Figure 1(a)). By dissection, we also found that body fat content including subcutaneous and intra-abdominal fat in obesity group was significantly higher than that in the control. The percentage for testosterone and perinephric fat in body weight of obese rats was >10%, while the percentage in normal rats was only <5%. The average level of serum triglyceride and total cholesterol in obesity group was more than 50% but 25% of normal group, respectively. After being fed with high-fat diet for 10 weeks, fasting blood glucose was significantly higher in GK rats than that in the normal control (23.6 ± 4.9 mmol/L versus 5.6 ± 1.46 mmol/L, *P* < 0.001).

### 3.2. Body Weight Changes of Rats after Different Surgeries

There was no difference in body weight among the groups before operation in normal rats. Weight loss existed in all the groups within 1 week after operation, of which weight loss in RYGB group was the greatest (*P* < 0.01). One week later, weight showed a gradual increase except that in RYGB group and MGBP group, and weight in RYGB group was significantly lower than MGBP group (*P* < 0.05). In 11th week after operation, only weight in RYGB group was still lower than the other groups (*P* < 0.05). In 15-16th week after operation, weight in SO and NC groups increased with the age growing, while weight in MGBP and SG groups began to decrease and was significantly lower than the other groups in 20th week after operation (*P* < 0.01) (Figure 1(b)).

In the obese rats group, there was also no difference in weight among the groups before operation. In 2nd week after operation, the average weight in RYBG, SG, and MGBP groups was significantly lower than that in the NC and SO groups, and weight loss in RYBG group was the greatest (*P* < 0.01). In 6th week after operation, weight in SG group was similar to that in NC group but decreased in 9th week after operation. In 12th week after operation, weight in RYBG and MGBP groups was significantly lower than that in NC group (*P* < 0.01), but there was no significant change in weight between SG group and NC group (*P* > 0.05, Figure 1(c)).

In GK rats, there was no difference in body weight among the groups before operation. Weight loss varied in all the groups within 1 week after operation. One week later, weight increased in NC and SO groups but continued to fall in the operation groups. The weight was NC, SO > SG, RYG, MGBP > TG (*P* < 0.01) on the 10th day after operation, NC, SO > SG, RYGB > TG, MGBP (*P* < 0.05) in the 2nd week after operation, and NC, SO > SG, RYGB, MGBP (*P* < 0.01), SG, RYGB, MGBP > TG (*P* < 0.01) in the 5th week after operation (Figure 1(d)).

### 3.3. Changes of Ghrelin, Obestatin Plasma Levels and the Ratio of Ghrelin/Obestatin

**Figure 2:**
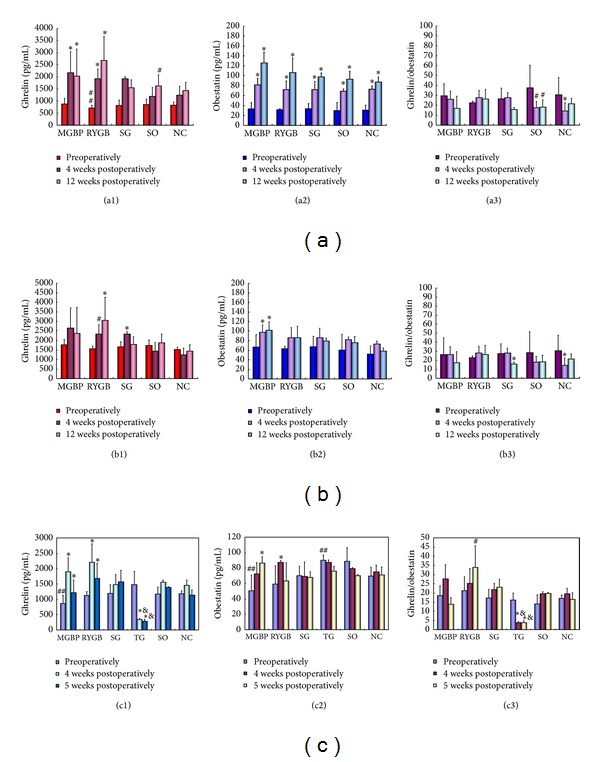
Changes of plasma ghrelin, obestatin and ghrelin/obestatin ratio after different operation treatments. (a) Ghrelin (a1), obestatin (a2) plasma levels and the ratio of ghrelin/obestatin (a3) in normal rats; (b) ghrelin (c1) and obestatin (c2) plasma levels and ghrelin/obestatin (c3) in obese rats; (c) ghrelin (d1) and obestatin (d2) plasma levels and ghrelin/obestatin (d3) in Goto-Kakisaki (GK) rats. ^##^Compared with NC group preoperatively, *P* < 0.05. ^#^Compared with the same group preoperatively, *P* < 0.05. *Compared with the same group preoperatively, *P* < 0.01. ^&^Compared with other groups preoperatively, *P* < 0.01. SG, sleeve gastrectomy; MGBP, minigastric bypass, RYGBP, roux-en-Y gastric bypass, SO, sham operation; NC, normal control; TG, total gastrectomy.

In normal rats before operation, ghrelin levels were significantly lower in RYGB group than those in NC group (*P* = 0.021), but no differences in obestatin levels and the ratios of ghrelin/obestatin were observed among all the groups. After operation, ghrelin levels in RYGB and MGBP groups significantly increased, but ghrelin levels in SG group only showed a significant increase in 12th week after operation. Obestatin level increased at different degrees in different groups after operation, but no statistical significance was present among groups when growth factors were eliminated through ANCOVA. Ratio of ghrelin/obestatin decreased in SO group (*P* < 0.05) after operation and in NC group 4 weeks after operation (*P* < 0.01). There was no significant difference when the data were corrected by ANCOVA (Figure 2(a)).

In obesity rats group, ghrelin, obestatin, and ghrelin/obestatin showed no difference among all the groups before operation but were higher than those in normal rats. Ghrelin levels significantly increased in RYGB group postoperatively (*P* < 0.05) and increased in SG group 4 weeks after operation (*P* < 0.01). Obestatin levels only significantly increased after operation in MGBP group (*P* < 0.01). Ghrelin/obestatin ratio decreased in SG group 12 weeks after operation (*P* < 0.05) and in NC group 4 weeks after operation (*P* < 0.01) compared with that before operation (Figure 2(b)).

In GK rats before operation, ghrelin plasma level in MGBP group was lower than that in NC group, while there was no difference among other groups. At 2 weeks postoperatively, ghrelin plasma level in all the groups except TG group was increased at different degree. At 5 weeks postoperatively, ghrelin plasma level in MGBP and RYGB groups was higher than that before operation. Ghrelin plasma level in TG group was significantly lower than the other groups and that before operation. In GK rats before operation, obestatin in MGBP group was lower than that in NC group (*P* = 0.033), while obestatin in TG group was higher than that in NC group (*P* = 0.041). After operation, obestatin plasma level in MGBP and RYGB groups was higher than that before operation. But there was no difference in obestatin levels among the groups. Preoperatively, ghrelin/obestatin ratio showed no difference among the groups. Ghrelin/obestatin ratio in TG group decreased significantly (*P* < 0.01) after operation. In 5 weeks after operation, RYGB group was higher than before operation (Figure 2(c)).

### 3.4. Correlation of Ghrelin, Obestatin, and Ghrelin/Obestatin Levels with Body Weight in Obese Rats or GK Rats

From Table 1, we can see that there was a positive relationship between ghrelin, obestatin plasma levels and body weight in normal rats, while there was a negative relationship between ghrelin/obestatin and body weight in both normal rats and obese rats. No correlation between ghrelin, obestatin plasma levels, ghrelin/obestatin, and body weight was present in GK rats.

## 4. Discussion

Our present study confirms the efficacy of bariatric surgery for the cure of obesity and T2DM rats. However, in obese rats, weigh loss in RYGBP group was similar to that in MGBP group, but larger than that in SG group. For GK rats, the obvious weight loss occurred in TG and RYGB groups. These findings suggest that participants assigned to RYGB are more likely to achieve remission of obesity and T2DM, which are in accordance with previous studies [19, 22]. Recent studies even suggest conversion from SG to RYGB when the weight regains [23, 24]. The results indicated that weight loss was significantly improved after conversion (mean loss percentage of excess of body weight, 64.6% versus 47.1%) and all reflux symptoms are eliminated without any medication at the end of the followup [25]. Besides, several studies also have demonstrated that body weight loss is a common sequela after total gastrectomy [26–29]. However, most studies investigated its treatment effect for patients with gastric cancer or combined with T2DM [30, 31]. In this study we found that TG was also effective for decreasing weight for T2DM rats without gastric cancer or obesity and glucose level was still lower than that before operation when rats restored the food intake (Supplementary Figure 1 in Supplementary Materials available online at http://dx.doi.org/10.1155/2014/569435).

To reveal the mechanism of surgical treatment, roles of gastrointestinal peptide hormones such as ghrelin and obestatin raised our concerns. Ghrelin is a 28-amino-acid, appetite-stimulating peptide hormone secreted predominantly by the food-deprived stomach in rats and humans [32]. Upregulated ghrelin expression stimulates appetite and food intake, downregulates energy expenditure, and conserves body fat, ultimately leading to weight gain [32, 33]. Besides, ghrelin suppresses insulin secretion in humans and promotes weight gain [34, 35]. Thus, strategies that are effective for weight loss may decrease ghrelin level. However, in this study, we only observed a decrease in ghrelin level after TG, but not others, indicating the other mechanisms may be present for RYGBP, MGBP, and SG to control weight.

Obestatin is a recently discovered 23-amino-acid peptide encoded by the ghrelin gene. Obestatin has been shown to suppress food intake and decrease gastric emptying, therefore antagonizing the orexigenic effect of ghrelin [36]. As previously reported, obestatin was decreased in obese subjects and a nutritional marker reflecting body adiposity and insulin resistance [37]. Therefore, regulation of obestatin level may be another mechanism for strategies that can modulate body weight and energy homeostasis. In our study, obestatin plasma level showed no difference between obese and normal rats but was lower in GK rats. After MGBP surgery in obese rats or RYGB surgery in GK rats, obestatin was increased, suggesting that MGBP and RYGB may exert weight control role via regulation of obestatin. However, there was a study reporting that obestatin concentrations did not change after RYGB surgery in obese people [12]. Thus, ghrelin/obestatin ratio is suggested to be used to assess the treatment effect of surgery. Roth et al. reported that ghrelin/obestatin ratio was lower after surgery than before (−14%, *P* = 0.017) [12]. In this study, we also found that ghrelin/obestatin ratio significantly decreased in SG and TG groups. These implied that SG may play important roles for weight loss via influencing ghrelin/obestatin ratio. Our correlation analysis further addressed the evaluation function of ghrelin/obestatin ratio because only ghrelin/obestatin ratio was negatively correlated with weight loss in obese rats, but not ghrelin and obestatin.

However, there are still some limitations in this study. Although the average weight of obese rats is higher than that of age-matched normal rats, the weight is similar between the two groups at 28 weeks old (Supplementary Figure 2), suggesting that obese rats have self-limiting weight. There is a difference in food habits between human and rats, which may lead to different surgical results between rats and human being. Thus, further human studies are still needed.

## 5. Conclusions 

RYGB and MGBP may be more suitable for obesity, but TG and RYGB are recommended for diabetes. Their underlying mechanism may be modulation of ghrelin decrease for TG, ghrelin increase for MGBP and RYGB, and ghrelin/obestatin ratio decrease for SG.

## Supplementary Material

Supplementary Figure 1. The fasting blood glucose level in each group at different time points. SG,sleeve gastrectomy; MGBP, minigastric bypass; RYGB, roux-en-Y gastric bypass; TG, total gastrectomy; SO, sham operation; NC, Normal control.
Supplementary Figure 2. The body weight of normal rats and obese rats at different time points.

## Figures and Tables

**Table 1:** Correlation of ghrelin, obestatin, and ghrelin/obestatin levels with body weight.

Groups	Ghrelin		Obestatin		Ghrelin/obestatin	
	*r*	*P*	*r*	*P*	*r*	*P*
Normal	0.530	0.042	0.809	<0.001	−0.584	0.022
Obesity	−0.081	0.773	0.153	0.587	−0.695	0.004
GK	0.093	0.714	−0.268	0.282	0.153	0.545

## List of Abbreviations

T2DM: Type 2 diabetes mellitus
RYGB: Roux-en-Y gastric bypass
MGB: Minigastric bypass
SG: Sleeve gastrectomy
BMI: Body mass index
SO: Sham operation
TG: Total gastrectomy
SD: Sprague-Dawley
GK: Goto-Kakisaki.
